# Memoir on Sprains

**Published:** 1828-05

**Authors:** M. A. Pelletier

**Affiliations:** Hospital Surgeon at Mans.


### SPRAINS.

*Memoir on Sprains.* By M. A. Pelletier, Hospital Surgeon at Mans.

It is to be regretted that, while cases presenting any thing rare or peculiar are generally told with minuteness, little or no notice is taken of diseases that are apparently slight, but which, from their great frequency, ought to be well known, and have some proper rules of treatment laid down. This particularly applies to the subject we have chosen, as *sprains* are thought to be curable by any-body, and quacks employ, to cure them, means that are sometimes dangerous.

To be precise, we would define sprains to be the forcible extension of the white fibrous tissues in the vicinity of joints, and serving them as means of union, such as ligaments, capsular fibres, and tendons, which for the most part act as ligaments. The causes of sprain may be distinguished into direct or determining, and indirect or predisposing. The direct causes generally are all efforts supported by the articulations, either in the exercise of different professions or in the different accidents to which men are liable. As these efforts must separate the articular surfaces, so they must have the effect of stretching the ligaments more or less. Therefore, those joints most exposed to great efforts are the most likely to be sprained, particularly if these joints do not possess a certain laxity, capable of permitting considerable movements without much drag on their ligaments. The scapulo-humeral joint, in consequence of possessing this laxity, although partly exposed to such accidents, is rarely a subject of sprain: on the contrary, the tibio-tarsal, radio-capsule joints, &c., whose articulating surfaces are closely united, give the most frequent examples of sprains. The tibio-tarsal articulation is particularly so, and in itself presents more cases of sprain than all the other joints of the body. It can equally well be conceived that the ligaments in these articulations most exposed to support violent efforts are most liable to sprain. So M. P. remarks, that he has found that, out of ten sprains of the tibio-tarsal articulation, eight at least affect the external ligaments; the foot having more disposition to adduction than to abduction.—Among the indirect causes, some are relative to the mode of life or profession of individuals, others to constitution. In the first class may be placed the custom of taking violent exercise, the use of high-heeled shoes, pattens, &c.: in the second class we find considerable fatness in conjunction with a lymphatic temperament, the one increasing the weight of the body, and the other relaxing the ligaments; scrofula, &c.

*The nature and character of Sprains.*—It would at first appear that so simple an accident, particularly when there is no tearing of the ligament, could hardly cause any troublesome symptoms, but theory teaches, and experience every day shows, that fibrous tissues, which could be divided by a sharp instrument without pain, are never submitted to violent distention without causing much pain, and symptoms more or less severe; but the vital properties being naturally very obscure in these tissues, they cannot immediately be exalted, so that some days often will elapse between the distention and the development of the inflammation caused by it. And such is precisely the source of the imprudencies which patients commit, in continuing to exercise a limb after it has suffered a sprain. Suppose a violent extension to have been applied to the foot, the patient experiences at the moment a severe pain in the distended ligaments. This soon ceases, and he resumes his exercise, which he had for a moment interrupted. After some time, the vital properties develop themselves gradually in the distended ligaments, and inflammation shows itself; the pain becomes intolerable, and, if the subject is scrofulous, the consequences may be most serious. M. Pelletier thinks that, from neglect, a sprain becomes a greater evil and more difficult of cure than a fracture.

*Diagnosis of Sprain.*—An unfailing method of discovering sprain is to put the limb in the situation it was at the time of the accident, and gradually and carefully to bend the joint in the same way. For example, if an external sprain of the foot is suspected, to carry it gradually into adduction; soon pain begins, which disappears on moving the joint the opposite way.

Sprain should never be considered as a trivial accident. It often happens that what at first appears simple shows in a few days very urgent and distressing symptoms, when neglected or improperly treated. The greatest precautions are necessary, and it is better to err on the safe side by too much than too little care. To establish the basis of a regular treatment of disease, it is indispensable to be acquainted with the progress of the disorder, and to mark precisely its different periods: this is particularly applicable in sprain, since its treatment varies much in its different stages, and what would be serviceable at one stage may be most improper at another. Let us suppose a sprain entirely left to nature's efforts, and going on as far as it can, we can distinguish four periods: 1st, the forcible extension of the ligaments, with severe but generally evanescent pains; 2d, the development of acute inflammation in the parts injured; 3d, the change from acute to chronic inflammation; 4th, organic degeneration of the inflamed parts, and accidents attending it. After describing each of these periods, we shall point out the appropriate treatment.

First period—the forcible extension of the ligaments. This begins at the instant when the accident has happened, and lasts generally from twenty-four hours to two or three days. It results from several causes. It may be asserted that, in general, inflammation manifests itself so much the more quickly as the extension has been great, the subject lymphatic, young, and gifted with much vitality: on the contrary, so much the more slowly as the sprain has been slight, the subject aged, or of a constitution less irritable. When sprain takes place, there is acute pain in the distended ligaments: sometimes this pain disappears very quickly, and the person resumes the exercise he was about. Now this is the insidious part of it, and favors the development of further mischief, by preventing persons from taking that rest which is so essential to the cure. Soon after the extension there comes on a puffy swelling in the neighbourhood of the parts affected, a true active œdema, sometimes even ecchymosis, greater or less. These must not be considered as the result of inflammation, (which has not had time to develop itself yet,) but as the effect of a lymphatic afflux into the cellular tissue where the pain was felt. What proves this is, that the swelling gives only an œdematous feel, without heat or local pain on pressure, and which quickly disappears with rest. Surgeons, who have not sufficiently observed this complaint, are deceived by this disappearance of the swelling, which they regard as the principal symptom; and look upon the disease as terminated when this is dispersed, that is, on the second or third day. So that they give up all further treatment, and permit exercise at the very time when inflammatory symptoms are about to appear.

Second period—development of active inflammation in the distended ligaments. The tissues of the living animal economy, when subject to the influence of excitation, *cæteris paribus*, inflame with a rapidity corresponding to their supply of blood-vessels, and to the degree with which they are endued with well-developed vital properties. But this inflammation is not immediate: it is, in fact, rare that it manifests itself before twelve or twenty-four hours in tissues the most disposed to its invasion; and, in those less disposed to its influence, many days elapse before it appears. This is in conformity with what happens after grand operations, particularly amputations of limbs, when numerous and different tissues are divided. We must not, then, be astonished that inflammation of fibrous tissues, whose vital properties are so obscure, does not appear till a distant period from the time of the accident producing it. This circumstance, attention to which is of the greatest moment in the treatment of sprains, is then quite naturally explained by the mode of organization of the affected tissues. Generally, inflammatory symptoms manifest themselves from the second to the fourth day, and very frequently when the first pain is quite gone. If there is little swelling, the parts sprained have a natural appearance, and the patient, not understanding his situation, often refuses to keep at rest. Spirituous applications appear to do good by helping to dissipate the swelling during this treacherous calm; but soon the scene changes. The patient feels in the injured part pain, at first dull, then more severe, at last acute, preventing the possibility of moving the joint without anguish. Soon after, a shining appearance, heat and swelling, painful feeling of distention, throbbings and dartings in the part, come on; and few medical men will not now acknowledge the necessity of rest and antiphlogistic means; but scarcely are these first symptoms diminished when the patient gets tired of confinement, and wishes to resume his ordinary avocations; while the surgeon, thinking he has now only to forward resolution, instead of feeling that there is still inflammation to destroy, abandons too soon the antiphlogistic system, and passes to a resolutory treatment, thus recalling the inflammatory symptoms, and making them chronic. Here terminates the second period. We may easily imagine what must be the consequence of applying in this stage spirituous resolving embrocations, &c. We have seen from it the inflammation rapidly extend itself to the synovial membranes and neighbouring tissues, bringing on disorganization and suppuration of these tissues, caries of the bones, hectic fever, and death.

Third period—change from active to chronic inflammation. The affected joint becomes the seat of an habitual irritation, too weak, when attended to, to bring back inflammation in the acute form, but sufficient to make it pass into the chronic. This joint then becomes enlarged, and towards night is swollen, feels stiff: the patient, not aware of the mischief that may follow, continues his exercise, and the more willingly as he looks upon the play of the joint as absolutely necessary to its gaining strength. We have seen cases kept up this way for years.

Fourth period—" Degeneration Lardacée," lardaceous degeneration of the inflamed white tissues. The continuance of chronic inflammation of the affected ligaments, the cure of which is the work of years, is of itself troublesome enough, and yet this is by no means the most fatal consequence that may occur from neglected sprain. Often, indeed, the chronic inflammation kept up by exercise and other means extends itself by degrees to the cellular tissues, the synovial membranes, periosteum, and even to the bones themselves. This inflammation becomes more intense according as it attacks parts more vascular and nervous, causing pains which daily aggravate till they force the patient to keep his bed entirely, and the surgeon to have recourse again to antiphlogistic means. But it is too late; the return of the lancinating pains announce a lardaceous degeneration, which, once established, there is no escaping from. The joint loses its natural form, puffs up, becomes round, the skin covering it becomes semi-transparent, and of a milky white; a constant feeling of deep distention, and at times violent lancinating pains, torment the patient. Often abscesses in the synovial membranes under the periosteum, denudation and caries of the bones, with fistulous openings. The lancinating pains become almost constant, with sleeplessness, marasmus, hectic fever, night sweats, colliquative diarrhœa, and, if the part is not sacrificed to the preservation of the individual, death is not slow in bringing to a conclusion this deplorable scene. Such are the terminations of sprains, neglected or empirically treated.

We now proceed to the *treatment*.

There are few diseases against which have been devised more numerous, much varied, and sometimes more opposite modes of cure. Must we infer from this that the authors who have mentioned them have been deceived, or that the disease may be cured by methods essentially different. The most opposite means may, we doubt not, cure a sprain; but this truth, which astonishes the ignorant man because he cannot understand it, which makes him consider the curative art as merely conjectural, and depending for its results upon chance, explains itself quite naturally to the physician, who reasons and draws his curative means from the result of observation.

We have already distinguished four periods in the history of this complaint, and the explanations we have added must make it clear that, if each period called for certain means, these means ought to be different when applied at these different periods. It is precisely for want of this important distinction, and from having considered sprains as always alike, whatever stage they might be in, that the same confidence has been extended to cold applications and emollient poultices, to leeches and stimulating applications, to Goulard water and camphorated spirit, rest and exercise, &c.; and that, in consequence, a perfect chaos has arisen from the *ensemble* of these means of cure. What is still more to be lamented is, that the use of certain means at particular periods being supported by the sanction of celebrated men, ignorant quacks employ the same means in all cases without distinction, and in all the stages of the disease. It is easy to conceive what mischief may arise from such abuses. There is but one way to avoid these fatal errors, and to establish fixed rules for the treatment of this disease, viz. to review the four periods as they occur, and to point out successively the treatment suitable to each. If the surgeon is called to a patient in the first period, when there is considerable pain caused by violent extension, there are two points to be attended to. The first is to examine if there is rupture of any of the ligaments, which might be determined by estimating the force of the cause, the situation of the parts at the time of the accident, the continuance of pains, and the rapid swelling of the neighbouring parts. This last symptom is important to note: it renders the development of inflammation almost inevitable, whatever precautions are adopted, and therefore at once indicates the necessity of antiphlogistic means. The second thing to be attended to is to allay the pains and prevent the accession of inflammation; and, among the means of doing so, *rest* is the first: without this all others will fail; and, in conjunction with rest, we must make use of *revulsive* or *resolutory means.* These are usually looked upon to consist in certain external applications, which have the power of hastening back the circulating fluids into the great vessels, from whence they had been sent with so much increased energy as to distend the capillary vessels of the part affected. Without cavilling about this definition, we shall distinguish these means into three classes essentially different in their manner of acting: 1st, cooling resolvents; 2d, astringent resolvents; 3d, irritating resolvents.

*Cooling resolvents.*—In this class are all those applications which diminish the heat of the part, as snow, cold bath, &c. It has long been recommended to plunge the affected part into very cold water immediately after the accident. Now this we can imagine may sometimes prove serviceable, but in others may be most pernicious. To satisfy ourselves of this, let us analyse its manner of action. If the action of cold is sufficiently strong, but only temporary, it causes at first a numbing of the part, a sort of local asphyxia; all the organic energy appears to come to the aid of the part threatened with mortification, and by a reaction, the force of which is in proportion to the strength and age of the patient, strongly awakens the life of that part, pushes towards it with force a great quantity of blood; it becomes, in fact, a centre of irritation, sometimes of instantaneous inflammation. One has only to plunge the hand into snow or very cold water, and observe the effects, to be satisfied of this. This means, then, when employed only for a short time, by bringing on powerful reaction, acts as a very active excitant. Some authors, for this reason, place heat and cold on the same parallel, calling the first a direct, the second an indirect excitant, their results being similar. If the action of cold is energetic and long continued, the parts submitted to its influence are as it were paralysed, and this suspension of their vital properties may cause the extinction of their irritation. This is what happens in a part frost-bitten. After all these considerations, one may perceive that the immersion must be, *cæteris paribus,* prolonged as long as reaction is to be feared; longer, consequently, in a young than an old subject, in a vigorous than in a weakened state. If one might fix a general period during which immersion should be continued, we would say from eight to twelve hours. We have adopted for the employment of this means a method which appears to obviate the greater part of the inconveniences which may attend it. It consists in plunging the affected part into water, the temperature of which is only a few degrees below that of the skin, and gradually diminishing the temperature till the requisite cold is induced. By doing this we almost always avoid the inflammatory reaction, which might show itself after twelve or fifteen hours cooling. There are circumstances which ought to prevent this means being used, as, for instance, in women near the menstrual period, in persons subject to internal inflammations, and in all immediately after meals, as we have seen it, when this was not attended to, cause violent vomitings.

*Astringent resolvents.*—This includes those applications which, applied topically, cause a constriction of the parts, without any apparent inflammatory reaction, as Goulard water. The best way of applying this is to keep the part constantly covered with compresses moistened with this fluid during several days. We would prefer this plan to the immersion.

*Irritating resolvents.*—By this is meant camphorated spirits, soap liniment, eau de Cologne; all, in fact, which have alcohol for their base. So far from being resolvent, we are inclined to think that their effect is to bring on inflammation where it was not before, and increase it if previously there. They should never be applied in the first or second periods; in the third they are sometimes applicable. Much mischief is done by the indiscriminate applications of such medicaments, and therefore they ought to be proscribed in these cases.

*Compression.*—Some writers have recommended the use of compression in the first period of this complaint, considering it as opposed to the tumefaction of the parts affected; but reason and experience demonstrate that this means is then most hurtful. It can only be useful when the patient is getting better, and beginning to take exercise.

*Narcotics.*—Any means capable of removing or even allaying the pain must be serviceable: in fact, the pain acts as an exciting cause to inflammation, which it is so essential to prevent. Narcotics joined with alcohol, such as the absurd and contradictory association of an irritating and calming medicament, like laudanum, are hurtful. A simple aqueous decoction of opium, or the poppy heads, or infusion of saffron, employed in fomentation or poultice, is generally successful in allaying pain.

*Local bleedings.*—The application of leeches is sometimes useful, sometimes even indispensable, in the treatment; sometimes, however, it is hurtful. For example, when the sprain is slight, the subject weak, of a dry nervous temperament, there is no need for them; the other means suffice: but if the sprain is violent, the subject young, vigorous, of a lymphatic or sanguineous temperament, and if the swelling immediately becomes great, then leeches must be freely had recourse to. They should always be applied to the most cellular and fleshy parts, and several inches away from the strained parts. When applied immediately to the injured parts, they constantly produce troublesome effects. The irritation caused by the bites unites with that from the sprain, the swelling increases frightfully, and the inflammation becomes more intense.

*Emollient applications.*—Under this head we range poultices, warm baths, fumigations, and fomentations. These means, the advantages of which are incontestable in the treatment of sprain, are often very successful after leeching. We have observed that, when they are applied before the leeches, and particularly when very hot, they increased the swelling, and promoted the development of inflammation, rendering the pains sometimes so intolerable that the further use of them becomes impossible. We should then always premise a copious local depletion, and regulate the temperature, so as not to increase the heat in the affected part, which is already too considerable.

*Treatment of the second period of Sprain, or acute inflammation of the distended ligaments.*—Although in the first stage many means, apparently opposite in their mode of action, have produced equally satisfactory effects, we must not expect this in the second. There now exists an inflammation, painful in its nature and difficult to root out, owing to the organization of the affected tissues, and dangerous in its consequences, by its contiguity to a joint which it may involve. Only one kind of treatment now remains, namely the antiphlogistic. Thus the cold bath, but particularly alcoholic preparations, may produce the most fatal consequences. To remove all causes capable of inciting or increasing inflammation,—to diminish the quantity of blood carried to the part inflamed,—to oppose, as much as possible, the exaltation of vital properties in the affected tissues,—to destroy, or at least lull, pain,—to avoid or oppose the irritation of organs which sympathise more directly with the diseased parts,—such are the principal indications and the basis of treatment at this period. The complete rest of the affected joint, the horizontal position to prevent swelling, leeches in abundance, and with the precautions above mentioned, and then large and thick poultices made of linseed meal and decoction of poppies, are to be had recourse to. If poultices keep up the heat too much, fomentations may be substituted. The intimate connexion between the intestinal canal and the joints is now well known: it is, therefore, necessary to put the patient on a mild regimen, to avoid whatever may possibly produce gastro-intestinal irritation. It is amply proved by experience that indigestion, the abuse of spirits, &c. have an evil influence on sprains.

*Treatment in the third period, when the inflammation has become chronic.*—When the patient has not attended to giving the joint rest for a sufficient time, or when the treatment has been improper, &c. the inflammation of the distended ligaments may, in moderate cases, insensibly diminish in intensity; but, being always kept up by improper treatment, or injudicious exercise, it at length passes to a chronic state, and may thus perpetuate itself indefinitely. Consulted under such circumstances, the surgeon must not lose sight of the fact that this chronic inflammation, although it does not offer any very pressing symptoms, is not the less an exaltation of vital properties, which it is necessary to oppose by appropriate means: therefore exercise, exciting resolvents, and all stimuli, of whatever nature they may be, are directly mischievous. It is by not attending to these principles that physicians prolong such cases during years; and, what is more distressing, cause, by the empirical applications made use of, a degeneration of the affected tissues, inflammation and caries of the bones, and even loss of limb. It is then essential, in this third period, to employ still the means recommended in the second, but modified according to the different form of the inflammation. Thus rest is not so essential, at least, unless the mischief is so advanced, and disorganization so much to be dreaded, that it only remains to attempt a cure by anchylosis, and then the limb must be fixed in the most convenient position. But, should this not be the case, the joint should daily be moved gently, taking care, as much as possible, during those movements, to place the affected ligament in a relaxed position, so as not to bring on again fresh distentions. Leeches may be applied with much advantage, if there is swelling and pain; five or six, every two or three days, will be sufficient. Here it is particularly necessary to apply a principle in pathology too little known. In chronic diseases the treatment should be chronic, —that is, should act in a slow but continued manner. It is by employing local bleedings in this manner for thirty or forty days that the most satisfactory results have been obtained, not only in sprains, but in almost all slow inflammations, even with threatening of disorganization. When the inflammation is quite dissipated, and the joint in an indolent state, and still a puffiness and soft feel in the ligaments remain, we may then have recourse to resolvents, but cautiously, and without ever painfully increasing the sensibility of the parts affected. Goulard water, hydrosulphureous baths, and light frictions with camphor and calomel, mixed with lard so as to make an ointment, moderate exercise, the part being properly supported by bandages and laced stockings, if it is the foot.

*Treatment of the fourth period, when there is disorganization of the inflamed parts, and other accidents.*—The further we advance in the different periods of this complaint, the treatment becomes the more difficult and uncertain. When chronic inflammation has disorganised the white tissues,—when the ligaments, tendons, and synovial sheaths are already converted into a white mass, homogeneous and lardaceous, there scarcely remains any hope either of restoring these parts, or of stopping the progress of this fatal disorganization. When this alteration, generally known by the name of white swelling, is recent, and the health of the person good, the case sometimes ends favorably; but, if it is of long standing and deep seated, with lancinating pains, and particularly if the patient be scrofulous, all attempts at cure are useless: we can only palliate. Many means have been recommended in this fourth stage, as any thing which has for its object to destroy a deep-seated irritation by a superficial one, either of the skin or subcutaneous cellular substance, such as ammoniacal poultices, spirituous liniments, blisters, setons, moxas, cauteries, &c. When these means have succeeded, and there no longer is deep-seated inflammation, and no disposition to relapse, even under these favorable circumstances, too strong applications have reproduced the deep-seated inflammation by the extension of the superficial one, and have hastened the progress of a disorganization which had perhaps been stopped in its course. Judge, then, what disorders these applications must occasion when applied to parts already inflamed and the seat of the white swelling. The pain speedily becomes insupportable, swelling and heat come on; sometimes suppuration in the interior of the joint, with hectic fever, &c. till death closes the scene. We have seen subjects in the flower of their age, and whose general health did not appear much changed, perish within twenty or thirty days from the time of applying these means. One must, then, never lose sight of these results, and dread them still, even when the joint appears the most favorably disposed to stand without injury the use of moxa, setons, blisters, &c., since the inflammation caused by these means may extend to the subjacent tissues, and there bring on the symptoms which have only been lulled.

If, then, there is any hope of cure, it is more prudent and certain to employ those means recommended in the third period, modifying them always according to the circumstances: rest, partial bleedings from the part, emollient and calming topical applications, and, lastly, gentle resolvents, ought to form the basis of the treatment. If these means will not do, no other will; the disease is incurable, and the complaint goes on from bad to worse, till he either dies or submits to an operation by which the affected parts are removed. But this amputation is not always practicable, either from the situation of the complaint, (as, for instance, when seated in the pelvic articulation, or in the vertebral canal,) and also from various circumstances, which may contra-indicate its employment. It may then be useful to point out some fundamental rules in those cases where we might save a patient by sacrificing a part for the preservation of the whole, and those in which the operation would only add new sufferings without the chance of benefit. Now, where the patient has had his constitution generally and extensively altered before the local accident which calls for the amputation, it would not only be useless to propose amputation, but it would do harm. This then is one case where we should not amputate.

When the patient has had pretty good health before the accident, and chronic suppuration, with sanious absorption which usually accompanies it, has brought on a constitutional derangement, "*cacochymie*," attended with dyspnœa, dry cough, especially when the patient makes a deep inspiration or the chest is struck upon, colliquative diarrhœa, slow fever, partial night sweats;—amputation under these circumstances might remove the first cause of the symptoms, but will not destroy the effects produced on the respiratory or digestive organs. These are not capable of retrograding, because they depend not only on the chronic inflammation of the tissues affected, but also on the organic lesions produced in these tissues by prolonged inflammation. This then is another case unfit for amputation.

When there is a decided scrofulous taint in the constitution, it should forbid the operation.

When a patient is originally of a sound constitution, and experiences a deep-seated chronic inflammation, with disorganization, in one of the principal joints, as the foot, knee, wrist, or elbow, in consequence of a local accident, such as a bruise, a wound, or a sprain, if that patient is nearly free from fever, the digestive organs in good order, the chest sound, and none of the internal viscera are affected, even although he should be exceedingly reduced in flesh and strength, we must not despair; for, by removing the affected part, we remove the focus of disease. This then is a case in which amputation is prudent and necessary, and such patients will recover sooner from amputation than those where they are in high health when the operation is performed; for, in the first case, the system being reduced to a kind of languor and inertness by its losses and sufferings, does not present those febrile and inflammatory reactions, which in the other may sometimes compromise the success of the operation, and cause a fatal termination.*

* Revue Medicale.

